# Glances in Immunology of HIV and HCV Infection

**DOI:** 10.1155/2012/434036

**Published:** 2012-06-07

**Authors:** Maria Giovanna Quaranta, Benedetta Mattioli, Stefano Vella

**Affiliations:** Department of Therapeutics and Medicines Evaluation, Istituto Superiore di Sanita', 00161 Rome, Italy

## Abstract

Since the identification of HIV and HCV much progress has been made in the understanding of their life cycle and interaction with the host immune system. Despite these viruses markedly differ in their virological properties and in their pathogenesis, they share many common features in their immune escape and survival strategy. Both viruses have developed sophisticated ways to subvert and antagonize host innate and adaptive immune responses. In the last years, much effort has been done in the study of the AIDS pathogenesis and in the development of efficient treatment strategies, and a fatal infection has been transformed in a potentially chronic pathology. Much of this knowledge is now being transferred in the HCV research field, especially in the development of new drugs, although a big difference still remains between the outcome of the two infections, being HCV eradicable after treatment, whereas HIV eradication remains at present unachievable due to the establishment of reservoirs. In this review, we present current knowledge on innate and adaptive immune recognition and activation during HIV and HCV mono-infections and evasion strategies. We also discuss the genetic associations between components of the immune system, the course of infection, and the outcome of the therapies.

## 1. Introduction

### 1.1. Human Immunodeficiency Virus Infection

Human immunodeficiency virus (HIV) is a retrovirus of the Lentiviridae family. This positive strand RNA virus infects specific cell populations of the immune system through its receptor specificity. At present, more than 33 million people are infected with HIV. HIV infection is characterized by an acute and a chronic phase, possibly leading to AIDS. Immediately after infection the viral load increases with exponential growth kinetics and CD4^+^ T cells rapidly decline [1, 2]. The peak of this growth curve coincides with the onset of a strong host immune response resulting in decreasing viral load and increasing number of circulating virus-specific CD4^+^ T cells. Then, the acute phase of HIV infection is accompanied by a selective and dramatic depletion of CD4^+^CCR5^+^ memory T cells predominantly from mucosal surfaces. This loss is largely irreversible and ultimately leads to the failure of the host immune defenses to clear the infection [3, 4]. This allows HIV to establish life-long latency and chronic infection.

Over the chronic phase of infection, the viral load remains stable, whereas CD4^+^ T cell levels gradually decline [5]. The chronic phase is clinically latent, but eventually without therapeutic intervention, the infection progresses to the symptomatic phase characterized by increased viral load and rapidly decreasing CD4^+^ T cell and also CD8^+^ T cell levels, making patients prone to opportunistic infections [6].

During HIV infection, both innate and adaptive immune responses are raised, but are insufficient or too late to eliminate the virus. Additionally, the very same cells and responses aimed at eliminating the virus seem to play deleterious roles by driving chronic immune activation that plays a central part in immunopathogenesis and progression to AIDS [7, 8].

### 1.2. Hepatitis C Virus Infection

 Hepatitis C virus (HCV) is a positive-stranded RNA virus belonging to the Flaviviridae family. Six major HCV genotypes have been identified and more than 100 subtypes have been identified through the world on the basis of molecular relatedness of conserved and non-conserved regions. Furthermore, several distinct but closely related HCV sequences coexist within each infected individual. These are referred to as quasi-species and reflect the high replication rate of the virus and the lack of a proofreading capacity of the RNA-dependent RNA polymerase.

More than 170 million people worldwide are chronically infected with HCV, which is a major cause of chronic hepatitis, cirrhosis, and hepatocellular carcinoma. Individuals infected with HCV have two possible outcomes of infection, clearance or persistent infection, determined by a complex set of virus-host interactions [9].

The majority of episodes of primary HCV infection are asymptomatic and infection is often detected incidentally at the time of routine health examinations or when donating blood. After initial exposure to HCV, 54–80% of infected person develop persistent viremia despite the generation of HCV-specific antibodies detected by ELISA and HCV-specific cellular immune responses [10–12]. This indicates that anti-viral immune response is functionally ineffective in the majority of exposed individuals. A correlation between syntomatic disease and viral clearance has been reported, possibly due to a more vigorous immune response, which also results in greater liver injury [11]. In chronic HCV infection, the liver is typically infiltrated by mononuclear cells including CD4^+^ and CD8^+^ T lymphocytes, B lymphocytes, as well as natural killer (NK) cells (CD56^+^CD3^−^) and NK T cells (CD56^+^CD3^+^).

Knowledge of the early immune response at the site of infection derives from recent studies on experimentally infected chimpanzees [13, 14], the only animal model used to study immune responses during the natural course of infection. Research into viral-host protein interactions has recently progressed rapidly with the development of an effective *in vitro* HCV culture system, which was previously unavailable [15–18].

### 1.3. HIV/HCV Coinfection

 Infection with HCV is the most common co-infection in people with HIV, and HCV is categorized as an HIV-related opportunistic illness [19]. Complications related to HIV/HCV co-infection are becoming an increasingly important medical issue. Owing to shared modes of transmission, as many as 30 to 40% of people with HIV may also be co-infected with HCV. HCV is approximately 10 times more infectious than HIV through percutaneous blood exposures. Though HCV is less likely than HIV to be transmitted sexually or from mother to baby, some studies have shown that the risk of sexual or perinatal (mother to baby) HCV transmission is higher in HIV^+^ individuals. Moreover, HIV/HCV co-infection is a significant problem among injection drug users (IDUs) as well as men who have sex with men (MSMs). The introduction in the mid-1990s of highly active antiretroviral therapy (HAART) for HIV has caused a sharp drop in the number of deaths from AIDS. This means that people with HIV are living longer. Therefore, if they are co-infected, the complications from HCV have more time to develop [20]. These complications (cirrhosis, liver cancer, end-stage liver disease) generally develop over 20–30 years. Liver disease from HCV is now the leading non-AIDS cause of death in the U.S. in co-infected individuals with HIV.

The ways in which co-infection with HIV and HCV affect the body are still poorly understood. Most studies indicate that HIV can worsen hepatitis C. HIV/HCV co-infection has been associated with a faster rate of hepatitis C disease progression, higher HCV viral loads, and a greater risk of developing severe liver damage. The impact of HCV on HIV disease is less clear, but a majority of studies suggest that hepatitis C does not accelerate HIV disease progression. Hepatitis C can affect HIV treatment by increasing the frequency of liver toxicity related to antiretroviral drugs. There is also the potential for interactions between drugs used to treat HIV and HCV infections. However, with careful medical monitoring, many co-infected people can be successfully treated for both HIV and HCV. Some experts believe it is better to begin HIV treatment first in order to control HIV replication and increase the CD4 count, since hepatitis C treatment works better in people with stronger immune systems [21]. Some research suggest that even after starting HIV treatment, CD4 counts do not increase as rapidly in co-infected people as in people with HIV alone. However, in people with early-stage HIV disease and advanced hepatitis C, it may be better to start HCV treatment first, so the liver can more easily handle HIV drugs since many HIV medications are metabolized by the liver and some can cause liver toxicity (hepatotoxicity).

## 2. Antiviral Immune Response

### 2.1. Innate Immune Responses

The innate immune system constitutes the first line of defense against invading pathogens and it functions during the early phase of infection, before the development of specific adaptive immunity. It is based on epithelial barriers, the complement system, and cells with phagocytotic and antigen presenting properties, such as granulocytes, macrophages, monocytes, Langerhans cells, dendritic cells (DCs), NK cells, and  *γδ*  T cells [22, 23]. It plays a part in early restriction of the virus and in shaping the adaptive immune response, but at the same time participates in the establishment and spread of infection. DCs and NK cells are vital mediators of the innate immune system and promote the development of adaptive immune responses [24]. DCs are of pivotal importance because they are among the earliest targets of HIV and are crucial for activating and conditioning virus-specific T cells, a process that is largely influenced by the preceding innate immune response [25]. Myeloid DCs (mDC)s are professional antigen presenting cells present in blood, skin, and mucosal tissues, whereas plasmacytoid DCs (pDCs) are located in blood and secondary lymphoid organs and play important roles in innate immune responses to viruses through the production of type I Interferons (IFN) [26].

Unlike adaptive immunity, the innate immune cells do not use T cell receptors; they are not major histocompatibility complex restricted; and they apparently lack memory, which is essential in vaccination. An early non specific protective response may limit microbial replication and dissemination, and allow adaptive immunity sufficient time to mount an effective protective response.

Innate immune responses to viral infections generally feature the induction of both cellular responses via NK cells, and antiviral proteins, notably IFN-*α*  and IFN-*β*, which generate an antiviral state [27]. The recent discovery of a fundamental pathogen recognition system which boosts innate immunity and drives the induction of type I IFN has dramatically advanced understanding of host responses to viral infection [28]. This system is based on pathogen-associated molecular patterns (PAMPs), which are recognized by specific PAMP receptors expressed in the host cell, initiating signals that ultimately induce the expression of antiviral effectors genes [29].

The receptors of the innate immune system that recognize PAMPs are called pattern-recognition receptors (PRRs). Among PRRs, the family of Toll-like receptors (TLRs) have been studied most extensively. TLRs are membrane bound receptors and 10 different TLRs have been identified in humans. Of those, viral recognition is primarily mediated by TLR9 recognizing DNA, as well as by TLRs 7/8 and TLR3 sensing single-stranded (ss) RNA and doublestranded (ds) RNA, respectively [30, 31]. In addition, C-type lectin receptors, such as DC-SIGN, Dectin-1, and mannose receptor, have emerged as cell surface PRRs that play important roles in induction of immune responses against various pathogens [32]. DC-SIGN, in particular, has been attributed essential roles as an adhesion receptor, in mediating interactions between DCs and T cells, and as a PRR inducing specific immune responses [33].

#### 2.1.1. HIV

The innate immune cells involved in protection of HIV infection are Langerhans cells in vaginal and foreskin epithelia [34, 35];  *γδ* ^+^  T cells in rectal and vaginal epithelia; and macrophages, DCs, and NK cells in the subepithelial tissues.

Interestingly, opposing roles for mDCs and pDCs in HIV infection have been described [36]. Whereas mDCs enhance HIV infection through capture and subsequent transmission of the virus, pDCs inhibit HIV replication in T cells through the antiviral activities of IFN-*α*  [37]. Since pDCs are the major producers of type I IFN, it has been suggested that abnormal migration and localization patterns of this important cell type may be a key defense strategy of HIV [38].

NK cells play a major role in preventing the early spread of viruses by producing cytokines and directly killing infected cells. NK cells may be crucial for early control of HIV infection and can have important roles in editing the function of DCs, thereby affecting their ability to prime antiviral effector T cells. They kill cells by releasing perforin and granzyme that cause the target cell to die by apoptosis. NK cells do not require activation in order to kill target cells, do not express T cell antigen receptors (TCR), whereas they express Fc receptor (FcR) to lyse cells through either to activate or to suppress their cytolytic activity. In general, NK receptors recognise missing self (MHC), induced self, or modified self (stress signals) proteins as their ligands [33].

As in NK cells,  *γδ*  T cells express the inhibitory receptors KIR, which recognize major histocompatibility complex class I molecules and inhibit cytotoxic responses [38]. The NKG2D activating receptors are also found on  *γδ*  T cells, and they recognize the human MHC class I chain-related proteins MICA and MICB.

The CC chemokines RANTES, MIP-1*α*, and MIP-1*β*  are produced by activation of macrophages, DCs, T cells, NK cells, and  *γδ*  T cells. These 3 CC chemokines can block the CCR5 coreceptors and prevent HIV infection *in vitro* [39]. There is *in vivo* evidence indicating that raised concentrations of CC chemokines downmodulate the cell surface expression of CCR5. An inverse correlation was established between the concentration of the 3 CC chemokines and the proportion of cells expressing CCR5 in macaques immunized with SIV gp120 and p27 [40].

*γδ*  T cells are involved in innate immunity and mucosal protection; they produce Th1 and Th2 types of cytokines [41], and they lyse HIV-infected target cells [39].  *γδ* ^+^  T cells also generate antiviral suppressor factors MIP-1*α*  (CCL3), MIP-1*β*  (CCL-4), and RANTES (CCL-5), which can prevent SIV infection by binding to and down modulating the CCR5 coreceptors [42].

Defensins have emerged as further components of innate immunity and may contribute to mucosal protection against HIV-1 infection especially that of oral mucosa [43].

Finally, important intracellular innate antiviral factors are represented by APOBEC3G (apolipoprotein B mRNA-editing, enzyme-catalytic polypeptide-like-3G), TRIM-5 (Tripartite motif-containing protein 5), and tetherin.

APOBEC is packaged into retroviral virions, and it deaminates viral cytidine to uridine, rendering them nonfunctional and inhibiting viral replication. APOBEC3G has also been found to inhibit HIV by an additional mechanism, possibly at or prior to the reverse transcription stage of the viral RNA [44]. This innate mechanism of resistance to retroviral infection is counteracted by the HIV-1 viral infectivity factor (Vif), which protects the virus by preventing incorporation of APOBEC3G into virions and by rapidly inducing its ubiquitination and proteasomal degradation [44].

Tetherin is a human cellular protein, an IFN-inducible factor of the innate immune system, which exerts antiviral activity against HIV-1 and other enveloped viruses by tethering nascent viral particles to the cell surface, thereby inhibiting viral release. In HIV-1 infection, the viral protein U (Vpu) counteracts this antiviral action by down-modulating tetherin from the cell surface [45].

TRIM5 is a protein encoded in humans by the TRIM5 gene [46]. The alpha isoform of this protein, TRIM5*α*, is a retrovirus restriction factor, which mediates species-specific early block to retrovirus infection. TRIM5*α*  was isolated as a rhesus macaque protein responsible for blocking infection by HIV-1. The human version of TRIM5*α*  does not target HIV-1, but can inhibit strains of the murine leukemia virus (MLV) as well as equine infectious anemia virus (EIAV) [47].

#### 2.1.2. HCV

While the majority of HCV-infected patients progress to chronic hepatitis, a small fraction of individuals are able to clear the virus. Resolution of infection occurs within the first few weeks of infection, suggesting that innate immune functions may be critical for early control. Although all nucleated mammalian cells are able to secrete type I IFN, the first response to HCV infection is thought to be IFN-*β*  production by infected hepatocytes. In hepatocyes, recognition of viral motifs at the cell surface is mediated via TLR3, while the retinoic acid-inducible gene (RIG)-I acts as a intracellular PAMP receptor for double-stranded (ds)RNA. These two independent signaling pathways are suggested to be involved in the activation of interferon regulatory factor (IRF)-3 and of nuclear factor kappa B (NF-*κ*B) leading to a transcriptional response that results in the secretion of IFN-*α*/*β*  from the infected cell. Activation of NF-*κ*B also induces the expression of proinflammatory cytokines and chemokines that function to amplify the inflammatory response and facilitate leucocyte recruitment to act in concert with IFN-*α*/*β*  in the host response to HCV [48].

 Secreted IFN-*α*/*β*  acts in both autocrine and paracrine pathways by binding IFN receptors to trigger activation of Jak-STAT pathway, leading to induction of IFN-stimulated genes (ISGs), which are the effectors of the host response to virus infection [49].

NK cells, which are present in greater numbers in the liver than in other organs, contribute to the pathogen-induced immune responses. Given the difficulties in generating efficient adaptive immune responses within the liver, the role of innate immune mechanisms in the induction of defensive responses is probably greater than in other tissues [50]. In the inflamed HCV-infected liver, activation of innate immune response induces NK cells to secrete IFN-*γ*  and to up regulate the expression of the chemokines CXCL9 and CXCL10. These chemokines bind CCR5 and CXCR3 on liver infiltrating lymphocytes and guide them into the space of Dissè and the parenchymal tissue. In addition, NK cells have a strong cytotoxic potential and are able to rapidly attack target cells without prior immunization.

### 2.2. Adaptive Immune Responses

Adaptive immunity refers to antigen-specific defense mechanisms, induced during the chronic phase of viral infection, designed to remove a specific antigen. There are two major branches of the adaptive immune responses: humoral immunity and cell-mediated immunity. The first involves the production of antibody molecules in response to an antigen and is mediated by B-lymphocytes; the second involves the production of cytotoxic T-lymphocytes (CTL), activated macrophages, activated NK cells, and cytokines in response to an antigen and is mediated by T-lymphocytes.

#### 2.2.1. HIV

Soon after infection, p24 antigen is detectable in serum and disappears as the person seroconverts, developing an antibody response to envelope and core antigens. Most of the humoral antibody response is directed at the viral envelope and has little or no neutralizing effect. Neutralizing antibodies are directed at particular epitopes within the variable loop regions of gp120 and the pre-fusion complex of gp41 [51].

CD8^+^ T cells mediated immunity is the most effective: upon recognition of viral antigen presented by the epitope-bearing MHC-I restricted molecule, CD8^+^ T cells become activated CTLs, killing the presenting cell by induction of apoptosis through the release of cytotoxic molecules as perforin and granzyme A/B, or by activating the Fas-ligand pathway [52]. CTL responses are detectable throughout the course of infection, generally being lost only late in the disease. These responses maintain significant pressure on viral replication and are important for the initial control of HIV infection and for the determination of viral set point. In general, the response is narrowly focused in the first weeks to months of infection and then broadens during the asymptomatic phase, prior to decreasing both in breadth and magnitude late in the disease.

In HIV infection, as in other viral infections, recognition of viral antigenic peptides activates a CD4^+^ T helper response, expressing a wide range of cytokines (including IL-2, IFN-*γ*  and tumor necrosis factor (TNF)-*β*) which co-ordinate a multi-cellular cell-mediated response against the invading virus. Activated proliferating HIV-specific CD4^+^ T cells are detectable in early infection. The virus infects activated cells more easily because they express high levels of the co-receptor CCR5 and also replicates more efficiently in proliferating cells. HIV-specific CD4^+^ T cells are preferentially infected early the disease process and subsequently become difficult to detect. Antigen-specific CD4^+^ T cells are detected at only low levels at other stages of infection, except in sub-populations of individuals capable of controlling their infection naturally, referred to as long-term non-progressors (LTNP). Similarly, the majority of HIV-specific CD4^+^ T cells detected are able to make IFN-*γ*  but not IL-2 [53]. This lack of appropriate CD4 help likely compromises CD8^+^ T cell responses and neutralizing antibody responses, especially those to new variants of the virus as they arise. Later in the disease, also CD4^+^ T cells recall responses to other pathogens and antigens are progressively lost, compromising immune responses to a range of pathogens.

Within the T cell compartment, much interest has been focused on an altered balance between proinflammatory Th17 cells and regulatory T cells (Tregs) in HIV infection. Th17 cells are CD4^+^ T cells that produce IL-17 and play a central role in host defence against bacterial, fungal, and viral infections at mucosal surfaces [54]. It has been recently reported that there is a significant loss of Th17 cells in the gastrointestinal tract of HIV-1 infected individuals [55]. Because IL-17 serves to maintain the integrity of the mucosal barrier, loss of Th17 cells may permit the increase in microbial translocation across the gastrointestinal mucosa that is observed in pathogenic lentiviral diseases. Recent studies suggest that the replenishment of Th17 CD4^+^ T cells in the gut mucosa during highly active antiretroviral therapy, or during nonpathogenic simian immunodeficiency virus infections in the nonhuman primate models, correlates with better restoration and function of the gut mucosal immune system [56].

Tregs represent a small subpopulation of T cells involved in preventing or inhibiting autoimmune and inflammatory disorders [57], but much controversy exists regarding the role of Tregs in HIV pathogenesis. One study demonstrated expansion of Tregs during HIV infection positively correlating with CD4^+^ T cell activation and rapid disease progression, indicating a detrimental role of Tregs in the immune control of HIV infection [58]. Tregs are major producers of transforming growth factor, (TGF-)  *α*, which promotes tissue fibrosis and limits immune reconstitution [59]. In direct contrast, however, other studies have reported decreased levels of Tregs in HIV-infected individuals [60], and in one study, depletion of Tregs in HIV infection was found to be associated with immune activation [61].

#### 2.2.2. HCV

 There is only limited evidence for a significant role for anti-HCV antibody responses in viral clearance, as recovery from primary infection does not correlate with HCV-specific antibody titers or levels of antibodies directed against the E1 and E2 glycoproteins [62, 63].

The humoral immune response in primary HCV infection is of low titer and, with exception of responses against the core protein, the generation of the response is delayed [64]. HCV-specific antibodies become detectable in the serum after the appearance of the cellular immune response and the subsequent increase in alanine transaminase (ALT). Another characteristic aspect of the humoral response against HCV infection is the restriction of antibodies to the IgG1 subclass, without the usual switching to IgG3 (or IgG4) subclasses that typically occurs with maturation of an antiviral humoral response [64]. Interestingly, there may be an association between the development of IgG2 antibodies and viral clearance. The IgG2 predominance has been linked to a Th1 bias in CD4^+^ T cell responses [65].

Direct evidence for a protective role of HCV-specific antibodies, derives from limited *in vivo* studies in which chimpanzees were protected against infection with an HCV inoculum that was neutralized with HCV-specific antibodies *in vitro* [66]. Thus far, the hypervariable region-1 (HVR-1) and other regions of the HCV-envelope glycoproteins that are thought to bind to the putative HCV receptor complex have been proposed as targets for neutralizing antibodies [67]. Mutations in the HVR-1 have been associated with the emergence of the quasispecies leading to escape from neutralization [68, 69]. This area of the genome is a region that undergoes a high rates of nucleotide substitution in acute HCV infection [70] implying that neutralizing antibodies do provide significant selective pressure on the quasispecies and hence arguing for the importance of this aspect of the humoral response in clearance.

Despite this circumstantial evidence for a role of neutralizing antibodies, individuals with hypogammaglobulinaemia clear the virus at a similar rate to the general population [71].

The decrease of viral titer coincides with the appearance of HCV-specific T cells and IFN-*γ*  expression in the liver [72], which suggests that viral clearance is T cell mediated. These events coincide with the induction of genes that encode proteins of the adaptive immune responses, such as MHC class II proteins, immunoproteasome subunits, and chemokines.

HCV-specific CD4^+^ T cells are essential in the generation of a successful HCV-specific immune response. At the time of clinical presentation and ALT elevation, vigorous proliferation of HCV-specific CD4^+^ T cells with concomitant IL-2 and IFN-*γ*  production is readily detectable in the blood of patients who later recover and clear the infection [73–75]. In contrast, lack of an HCV-specific CD4^+^ T cell response or failure to maintain it for a sufficient time, particularly in the face of viral mutations or quasi-species shifts, is associated with development of persistent infection and chronic hepatitis [73].

As with CD4^+^ T cell responses, a strong multispecific CD8^+^ T cell response produced early in infection is associated with viral clearance [76–80]. CD8^+^ T cell responses directed against multiple epitopes spanning the HCV polyprotein were seen in chimpanzees that resolved infection, whereas animals that developed chronic infection generated narrow responses [80].

During acute HCV infection, CD8^+^ T cells appear functionally impaired, with reduced proliferation, IFN-*γ*  production, and cytotoxicity [76, 81, 82] and increased levels of programmed death (PD)-1 [83]. However, the dysfunction of HCV-specific CD8^+^ T cells resolves, and IL-7 receptor  *α*-positive (i.e., CD127^+^) memory CD8^+^ T cells become detectable, as soon as HCV-specific CD4^+^ T cell responses develop and the HCV titer decreases [76, 82, 84].

## 3. Mechanisms of Immune Evasion

To replicate and spread successfully, viruses have evolved a number of strategies to evade host defenses. These include escape from T-cell recognition, resistance to immunological effectors functions, and active subversion of the immune response. Because of the high replication rate and the lack of proofreading capacity of both HIV and HCV DNA polymerases, the sequence and immunogenicity of the main viral populations can rapidly change and provide means to escape from emerging humoral and cellular immune responses.

### 3.1. HIV

The envelope of HIV is the primary target for humoral responses, and the virus has developed numerous mechanisms for avoiding the effect of neutralizing antibodies that are primarily directed towards envelope protein epitopes. Firstly, many of the neutralizing epitopes are cryptic, hidden within the protein structure of the molecule, and exposed only transiently. To be effective at these sites, antibodies require a high affinity to compete with natural ligands. Secondly, major neutralizing epitopes are protected by protein glycans, which form a shield to provide steric hindrance against anti-gp120 interaction [85]. Finally, the glycoproteins are highly mutable, conferring upon virus the ability to mutate rapidly away from effective neutralizing antibodies with minimal cost to viral fitness. Resistance to antibodies by point mutation on the V2/V3 loop and N-linked carbohydrate glycans on gp120 has been indicated as hindering antibody neutralization [86].

Similarly, the virus has developed multiple mechanisms to avoid recognition by CD8^+^ CTL; these include both mutational and non-mutational mechanisms. Viral proteins, including Nef, Tat, and Vpu, interfere with antigen presentation by downregulating the expression of MHC-1 molecules on the surface of these cells [87]. Most of these act at a post-transcriptional site. Conversely, selective upregulation of HLA-C and E by Nef may protect infected cells from NK cell attack. Virus can mutate at targeted epitopes to avoid MHC restricted recognition, by decreasing binding affinity of the epitope to the presenting protein MHC-1 [88, 89]. Alternatively, mutations may alter the recognition of antigen peptide by a particular TCR, either resulting in no activation of the T cell or delivering an antagonistic signal to the T cell, preventing normal activation [90]. Mutations may also alter the chemistry of antigen processing through the proteasome and peptide-loading complex, so that peptide is no longer presented. Recent studies have indicated the expression of cryptic epitopes from proteins expressed through alternative reading frames.

Successful elimination of the virus requires a robust innate immune response and efficient priming of adaptive immunity. DC, are central in both these process. Evidences indicate that DC function is impaired during HIV-1 infection, contributing to a lack of effective antiviral adaptive immunity. This is either due to direct viral interactions with DCs or a result of indirect mechanisms, such as the production of IL-10 by monocytes during infection. pDCs activated by HIV-1 produce type I IFNs, which, in addition to inhibiting viral replication, may contribute to bystander CD4^+^ T cell death. Furthermore, evidence shows that pDCs produce T cell-attracting chemokines, which may facilitate viral spread by providing a source of new T cells for HIV to infect. Finally, HIV-exposed pDCs prime regulatory T cells, which could impair mDC function and block effector T cell activity, further blunting adaptive immunity [91, 92].

### 3.2. HCV

HCV, after the initial interaction with the host immune system, uses several mechanisms to nullify the selective immunological pressure during the later phases of infection, including the alteration of its antigenic epitopes to escape immune surveillance. The human immune responses to HCV and the countermeasures of the virus are directly relevant to antiviral therapy given the main use of IFN-*α*  in standard treatment. The best understood immune-evasive strategies include inhibition of protein kinase R (PKR) by the viral NS5A and E2 proteins [93], cleavage of cellular signal transduction molecules downstream of RIG-1 and TLR3 by the NS3/4A protein, and altering IL-12 production through secretion of the core protein.

Another evading strategy of HCV targets TLR7 expression, mRNA stability, and signalling. Recent studies have identified a significantly decreased TLR7 expression in the presence of HCV, both *in vitro* and *in vivo*. It was suggested that HCV may directly interfere with the transcriptional regulation of TLR7 mRNA. Despite decreased TLR7 expression in HCV-replicating cells, enhanced activation of IRF-7 was observed [94].

Many researchers have explored the hypothesis that the failure of HCV-infected individuals to mount an effective T cell response, thus developing chronic HCV infection, was due to the impairment of DC function [95, 96]. However, in spite of extensive studies, there is little overall consensus. The majority of studies observed decreased frequencies of both mDC and pDC during chronic infection [97–102]. It has been shown that the numbers of mDC and pDC in the liver of patients with chronic HCV infection were markedly increased, as compared to controls. Thus, it is difficult to determine if accumulation of DCs in the liver is casually related to the decrease of DC number in peripheral blood.

There is less agreement about the effect of chronic HCV infection on the function of circulating mDC and pDC. mDC from patients with chronic HCV infection have been reported to be inefficient at driving naïve T cell proliferation in allogeneic mixed-lymphocyte reaction (MLR) [97, 98, 101, 103]. However, in other studies, the ability to induce T cell proliferation in MLR or antigen-specific T cell responses was intact in DC obtained from patients with HCV [102, 104, 105]. A common finding in many studies is that IL-12 secretion by mDC is defective in cells obtained from patients with chronic HCV infection, while secretion of IL-10 increased as compared to healthy individuals [98, 99, 101, 106].

Many studies also described the impaired function of pDC in patients with HCV, resulting in abnormal IFN-*α*  secretion in chronic HCV infection [106].

The impairment of DC function may not only influence T cell priming but may also preclude productive cross-talk between NK and DC. The abnormality of the NKG2A-expressing subpopulation also affects DC/NK cross-talk during chronic HCV infection [50].

Cross-sectional studies have indicated that NK cells are perturbed in chronic HCV infection. There may be a decrease in the number of circulating NK cells and skewing of NK cell subset distribution toward increased numbers of the cytokine-producing CD56^bright^ population, relative to the cytotoxic CD56^dim^ subpopulation. In addition, recent studies have shown that binding of recombinant HCV E2 protein and crosslinking of CD81 inhibit NK cell functions, such as cytotoxicity, proliferation, and IFN-*γ*  and TNF-*α*  secretion *in vitro* [50, 107–109].

## 4. Polymorphisms in Host Response Genes

### 4.1. HIV

Epidemiological studies of genetic polymorphisms in human populations have enabled new ways to understand antiviral response and immune activation during HIV infection. The natural course of HIV infection is characterized by considerable variations among infected individuals, and is related to both viral and host factors, including genetic differences.

One of the first studies addressing this question was the description of almost complete protection from HIV infection conferred by homozygosity of a 32 base deletion in CCR5 (CCR5-l32) or a mutation in CCR2 (CCR2-64I) [110]. They provide strong protection and are found more frequently in LTNP than in rapid progressors.

Moreover, certain HLA alleles are associated with control of virus replication and slower progression to AIDS [111]. For virus replication control, a strong CTL response is crucial. CTLs are activated by binding to antigenic peptides presented by HLA, initiating the immune response. Many studies report the effect of HLA polymorphisms on the progression of AIDS [112, 113]. Many HLA alleles have been correlated with rapid disease progression, as HLA-A24, -A29, -B35, -C4, -DR1, and -DR3, whereas patients carrying HLA-B14, -B27, -B57, -C8, or -DR6 appear to develop AIDS more slowly [114, 115]. It is known that LTNPs remain asyntomatic and clinically healthy for more than 10 years without therapy [116, 117]. A number of studies [118–120] have shown a higher frequency of the HLA-B27 and HLA-B57 alleles in this group of patients, suggesting also a role of these alleles in the pathogenesis of HIV [111]. It has been shown that CD8^+^ T cells of individuals expressing HLA-B27 or -B57, targeted a defined and highly conserved region within the HIV-1 p24 Gag (amino acids 240 to 272) early in infection, and responses against this region contributed over 35% to the total HIV-1-specific T cell responses in these individuals. In contrast, this region was rarely recognized in individuals expressing HLA alleles associated with rapid disease progression [121].

The HLA genotyping could help identifying those patients with either a mild or aggressive course of their natural HIV-1 infection. This might influence the timing to start HIV therapy and allow a risk stratification that could be relevant in countries with limited access to antiviral drugs. Moreover, under antiretroviral therapy, HLA type may influence the risk of developing drug-resistant viruses, and therefore address the choice of drug regimens in naïve patients. Finally, since HLA loci vary by ethnic groups and regions, it may affect the response to specific vaccines in different parts of the world.

Recently, the association between polymorphisms in the inflammasome component NLRP3 and susceptibility to HIV infection has been demonstrated and adds to other studies linking inflammasome activation and IL-1/IL-18 production with HIV pathogenesis [122].

For the association of TLRs polymorphisms and HIV-induced inflammation, somewhat more insight exists. One study reported an association between two single nucleotide polymorphisms (SNP)s in TLR9 and rapid HIV progression as measured by CD4^+^ T cell decline [123], although the investigators did not evaluate the precise effect of these SNPs on TLR9 signalling. In contrast, a different TLR9 polymorphism has been linked to slow disease progression and found less frequently among individuals with high viral set point [124]. In addition, a frequent functional TLR7 polymorphism resulting in significantly less IFN-*α*  production has been associated with accelerated disease progression and may also be associated with increased HIV susceptibility, since this mutation was present more frequently in patients than in controls [125]. The importance of TLR7 signalling was further supported by a recent article demonstrating sex differences in the TLR7-mediated response of pDCs to HIV: pDCs from women produce markedly more IFN-*α*  in response to HIV-derived TLR7/8 ligands than pDCs from men, resulting in a higher degree of immune activation in women for a given viral load [126]. At the genetic level, this may be explained by the fact that TLR7 is X-linked and therefore women may have higher expression of this receptor due to unbalanced X-inactivation. Clinically, the more robust IFN-*α*  response in women is translated into women exhibiting lower viral loads early in infection but progressing faster to AIDS for any given viral load [127]. Taken together, these studies support the idea of type I IFN having dual functions, including antiviral activities and immune activation.

### 4.2. HCV

Combination therapy with Peg-IFN plus ribavirin is the standard treatment for patients with chronic HCV infection [128]. However, it eradicates HCV in only about half of the patients infected with genotype 1. The outcome of IFN-based therapy for HCV is dependent on the genetic systems of both the human host and the virus. Host genetic factors that influence the outcome of therapy include gender, race, and variation in genes of the immune system.

The dominant role of the immune-modulator IFN-*α*  in treating HCV infection has driven an extensive search for genetic associations between components of the immune system and outcome of therapy [129–131].

To date, the most prominent human genetic associations with outcome of therapy are SNPs within the IL28B gene. The strongest association was found for SNP rs12979860, located about 3 kb upstream of the IL28B coding region. Patients with a CC genotype at this SNP were more than twice as likely to achieve sustained virologic response (SVR) as patients with a CT or TT genotype [132]. Suppiah et al. [133] estimated that the cumulative effect of the favorable allele at the IL28B locus is to increase SVR by 32% relative to a population in which the allele is absent. The favorable association of the CC genotype was found in patients of both European and African-American descent, and differential prevalence of the CC genotype explained approximately half of the twofold poorer response rate to treatment found in African Americans. Several reports have associated failure of response also with the presence of SNP rs8099917 and rs12980275. All these associations were shown to be significant for genotypes 1 and 4 but not for genotypes 2 and 3 [134, 135].

The IL28B gene encodes the type 3 IFN, namely, IFN*λ*  that has been shown to inhibit HCV replication; so the variations in the IL28B gene mutations presumably affect the efficacy of the innate immune response against HCV during therapy [134].

Interestingly, the pretreatment expression levels of genes involved in IFN-stimulated genes (ISGs) have been found to be related to treatment outcome. The allele OASL rs12819210 constitutes an important independent factor that is significantly associated with SVR in peg-IFN-plus-ribavirin treatment, notably improving the predictive value of IL-28B rs12979860. In addition to OASL and IL-28B, the IFIT1 rs304478 A/A genotype favors a better therapy outcome, especially in patients with HCV-1 [136].

The most common associations with clearance after primary infection has been with the class II alleles, DQB1*0301, and DRB1*1101 which are in close linkage disequilibrium. It has been demonstrated that immunodominant HCV epitopes are presented by DRB1*1101 [137] and that T cell lines recognize HCV peptides presented by DQB1*0301 [138]. Similarly, another allele that has been associated with HCV clearance, DRB1*0101, was associated with a greater magnitude and broader range of epitope responses in an HCV-specific T cell proliferation assay [139].

By contrast, only very few of class I HLA allele associations have been described in association with clearance of HCV viremia. KIR2DL3 and its ligand, HLA-C1 has been associated with an increased likelihood of spontaneous and treatment-induced HCV clearance [140–142]. It has been postulated that this gene combination is protective because the KIR2DL3 binds HLA-C with a lower avidity than other inhibitory KIR and thus NK cells expressing this specific inhibitory receptor have a lower threshold for activation [143, 144].

NK cells are also inhibited by the heterodimeric receptor CD94 : NKG2A, which has the oligomorphic MHC class I molecule HLA-E as ligand. In a genetic study, homozygosity for the HLA-E^R^ allele has been shown to be protective against chronic infection with HCV genotypes 2 and 3 [145]. This protective effect was thought to be due to an effect on HLA-E-restricted T cells, although the HLA-E^R^ allele may have a lower affinity for peptides and hence be expressed at lower levels. This could, therefore, lead to less inhibition of NK cells via the CD94 : NKG2A receptor [146]. The presence of HLA-B27 allele has also been associated with viral clearance [147] and a functional mechanism for this effect has been reported with the recognition of an HLA-B27 restricted CD8 epitope [148].

Genetic associations with outcome of therapy have also been reported for the genes encoding KIR2DL5, IL-6, IL-12B, IL-18, CCL5, TNF-*α*, IFN-*γ*, osteopontin, GNB3, and CTLA4. Several SNPs of TLR, relevant adaptor molecules and cytokines mediated by TLR signaling can affect various clinical outcomes in Caucasian patients with chronic HCV. However, the role of these polymorphisms in acute infection has not been elucidated [149]. However, other studies have failed to find associations with many of these genes. The inconsistencies in these data appear to be due to differences among the studies in racial make-up and HIV co-infection status of the cohorts, the definition of therapeutic response, the therapy employed (IFN-*α*  monotherapy, IFN plus ribavirin, or pegylated IFN plus ribavirin), and the HCV genotype infecting the study participants.

Other immunogenetic polymorphism have recently been reported to influence outcome of HCV infection such as polymorphisms of the promoter region of the IL-10 gene with the IL-10-592 AA genotype associated with self limiting infection, and the IL-10-1082GG genotype associated with persistent infection [150, 151]. Another study demonstrated an association between the IL-10 ATA haplotype (IL-10-1082A, -819T, -592A) and spontaneous HCV clearance [151]. The authors postulated that the reduced production of IL-10 associated with this haplotype may result in a Th1-polarized CD4^+^T cell response, which may be associated with enhanced viral elimination.

The presence of polymorphisms of the TGF-*β*1 gene promoter that reduce expression of TGF-*β*1 is also associated with increased rates of clearance of HCV infection [152].

Simultaneous genotyping for all these SNPs could be a useful tool for individualizing antiviral therapy for chronic hepatitis C. 

## 5. Conclusions

HIV and HCV share many characteristics: both are RNA viruses and have similar blood-to-blood transmission routes. Despite these viruses markedly differ in their virological properties and in their pathogenesis, they share many common features in their immune escape and survival strategy.

In the last years, much effort has been done on the study of the AIDS pathogenesis and on the development of efficient treatment strategies and a fatal infection has been transformed in a potentially chronic pathology. Much of this knowledge is now being transferred in the HCV research field, especially in the development of new drugs, although a big difference still remains between the outcome of the two infections, being HCV eradicable after treatment, whereas HIV eradication remains at present unachievable due to the establishment of reservoirs.

In the era of effective HIV therapy, chronic HCV infection is a leading cause of liver disease and mortality in HIV-infected patients. Whereas treatment of HIV with ART appears to slow the progression of liver disease, co-infected patients remain at greater risk for HCV disease progression than patients with HCV mono-infection. Accordingly, effective HCV treatment is a priority in this population.

The knowledge of the immunogenetic background may enable to calculate a composite genetic risk, which will be of increasing importance in the tailored management of HIV and HCV mono-infected or co-infected patients. Obtaining a molecular understanding of the mechanisms by which the genetic associations modulate the outcome of therapy would substantially improve therapy. In addition, the immunologic characterization of the patients could be useful to speculate on the need for immunotherapy for these patients.
